# Firearm-Related Upper-Limb Injuries in Children: An 8-Year Single Institution Analysis

**DOI:** 10.1016/j.jhsg.2026.100968

**Published:** 2026-02-26

**Authors:** Julie Mekhail, Rhiana Rivas, Devin A. Maez, Patrick P. Bosch, Nikalus G. Skipp, Deana M. Mercer

**Affiliations:** ∗ Department of Orthopaedic Surgery, University of New Mexico School of Medicine, University of New Mexico, Albuquerque, NM

**Keywords:** Hand injuries, Orthopedics, Pediatric gunshot wounds (GSWs), Pediatrics, Upper-limb injuries

## Abstract

**Purpose:**

Pediatric firearm-related injuries are rising, reflecting an ongoing public health concern and burden on the health care system. However, studies examining regional gunshot wound (GSW) trends among children are scarce. We evaluated upper-limb injuries from GSWs at our state’s sole level-1 trauma center over 8 years. We hypothesize there would be an increase in upper limb ballistic injuries in our pediatric population and that understanding these trends may lead to improved supportive care and interventions aimed at mitigating future occurrences.

**Methods:**

A retrospective chart review of pediatric patients (age <18) referred for GSW-related injuries to the upper extremity from 2016 to 2023 was conducted. Data collected included patient demographics, injury characteristics, fracture locations, surgical management, and long-term complications. Trends and disparities were analyzed.

**Results:**

Of the 296 total patients with nonfatal pediatric firearm injuries, 58 patients sustained GSW-related to the upper limb (19.6%). Forty-two of these 58 patients (68.9%) had fractures. Most patients were men (79.3%) and Hispanic/Latino (65.5%) with a mean age of 15.2 years (range 6–17 years). The phalanx (*n* = 11) and humerus (*n* = 10) were the most commonly fractured bones. 62.1% (*n* = 36) of patients required operative management, 20 of which involved fracture fixation. Antibiotic administration was noted in 86.2% of patients, predominantly cefazolin. We observed primary sequelae, which included 15 nerve injuries and 42 fractures. Documented soft tissue infections occurred in 9.4% of patients. Over 37% of patients lived in impoverished areas.

**Conclusions:**

Pediatric gunshot injuries remain a notable concern with broad implications for medical care and public health. We identified a total of 296 pediatric patients with nonfatal firearm-related injuries in 8 years, 58 of which involved injuries to the upper limb. Orthopedic surgeons have a role in recognizing this epidemic, improving treatment protocols, and providing guidance for practical improvements in education in injury prevention.

**Type of study/level of evidence:**

IV (case series)

Firearm-related trauma is the second leading cause of traumatic pediatric death in the United States, second only to motor vehicle collisions.^1^ In nonfatal gun violence, a substantial proportion of GSW-related injuries involve the extremities and result in ballistic fractures that necessitate orthopedic treatment. A considerable increase in orthopedic burden exists because of the rising incidence of firearm injuries among children.^2^

Our state’s sole level-1 trauma center, located in the southwest United States (US), has observed a troubling rise in recent pediatric gunshot injuries.^3^ The state exhibits a high gun-per-capita rate of firearm ownership, with 55,340 firearms per 100,000 residents (one gun per every 1.8 people), reflecting geographic disparities that may be contributing to this epidemic.^4^, ^5^, ^6^, ^7^ Furthermore, the estimated cost of gun violence to the US health care system is $170 billion per year, including direct medical expenses and long-term costs associated with rehabilitation therapy and other ongoing complications.^8^ These staggering costs emphasize the additional economic burden on medical establishments, insurance companies, and taxpayers, highlighting the importance of investigating and addressing these issues swiftly and diligently.^9^^,^^10^ Understanding these dynamics is crucial for developing targeted strategies to mitigate the impact of firearm-related trauma on pediatric patients in our region.

Upper extremity (UE) and hand GSWs often result in complex fracture patterns, soft tissue damage, and neurovascular injury. Ballistic fractures can also have an extensive zone of comminution and are challenging to manage.^11^ However, epidemiological studies investigating trends in upper limb (UL) GSWs among the pediatric population are scarce. Therefore, our study focuses on reviewing the trends of these injuries and the associated clinical burden at our southwestern level-1 trauma center. We hypothesize that the rates of children presenting with UL GSW injuries steadily increase during our study period and that further analysis of these trends could contribute valuable insights to the existing literature. Reporting on these injury patterns is crucial to understanding the true scope of the larger firearm epidemic, and doing so may contribute to improvements in treatment protocols and inform prevention and advocacy efforts.

## Materials and Methods

Institutional review board approval for data collection and analysis (protocol no. 24-364) was obtained, similar to our previous study.^3^ All electronic medical records for patients diagnosed with gunshot injuries presenting at a tertiary level-1 trauma center in our state’s largest city from January 1, 2016, to December 31, 2023, were queried using International Classification 10th Edition (ICD-10) codes. This study period was selected to minimize limitations when using data spanning the ICD-9 and ICD-10 switch on October 1, 2015. The ICD-10 code list is depicted in the appendix (Table S1, available online on the Journal’s website at https://www.jhsgo.org). This coding system generated a list of pediatric patients aged 0–17 who presented to a level-1 trauma center, the only institution of this acuity in the state.

The area of interest was the greater urban area in which our hospital is located and, to a lesser extent, the entirety of the state, as many patients from across the region are transferred to this level-1 trauma center for higher levels of care.

**Figure 1:**
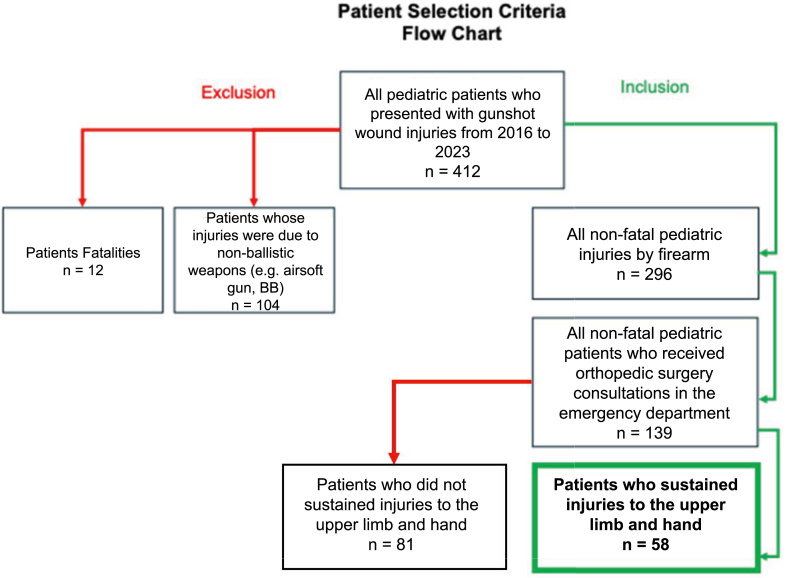
Flowchart of patient inclusion and exclusion criteria.

A retrospective chart review was performed for all patients identified meeting the following inclusion criteria: all nonfatal injuries by firearm, who received hand surgery consultation in the emergency department (ED) and who had injuries to the UL and hand. Detailed inclusion and exclusion criteria are diagrammed in the Figure 1.

Each patient’s electronic medical records were reviewed retrospectively in depth. Basic information from each chart was recorded, including date of injury, age at time of injury, sex, race/ethnicity, anatomic location(s) of projectile injury, and projectile type. Trips to the ED as opposed to outpatient presentations, orthopedic consults, and operating room visits, if applicable, were also recorded, including the specialty of the attending surgeon (eg, general surgeon, orthopedics, neurosurgery). Additionally, the location of the injury, bones fractured (if applicable), and treatment with an antibiotic course (including the medication name, dose, and length of treatment in days) and tetanus vaccine administration were noted, as well as outpatient follow-up. Injuries were classified into primary sequelae (eg, fractures, nerve injuries) and secondary sequelae (eg, infections, nonunions). Finally, in addition to clinical data, the patient’s zip codes were recorded to identify potential trends related to socioeconomic status and the risk of sustaining GSW injuries within our community.

Statistical analysis was then performed comparing the demographic data of patients included in our study to the state’s demographic data from the 2020 US census reported on census.gov using a chi-squared test. A *P* value was computed to determine a link, or lack thereof, between our data and the demographic data of the state. All trends were analyzed using a Mann-Kendall trend test. Kendall’s Tau was obtained to determine the strength and direction of trends, and a *P* value was obtained to determine statistical significance. A *P* value of < .05 was considered statistically significant for all statistical analyses.

## Results

Four hundred and twelve patients under the age of 18 who presented to our institution’s ED with a GSW in any anatomical location were identified by ICD-10 code. After excluding 12 fatalities and 104 BB/airsoft injuries, 296 patients met the initial inclusion criteria and 139 of these patients received an hand surgery consultation based on the characteristics of the injury as determined by the emergency medicine provider. Of these patients, 58 were determined to have gunshot wounds to the UE (Fig. 1). All patients received initial irrigation and debridement in the ED upon admission, along with temporary closure and splinting if needed. Mean follow-up for the cohort was 5.3 months.

**Figure 2:**
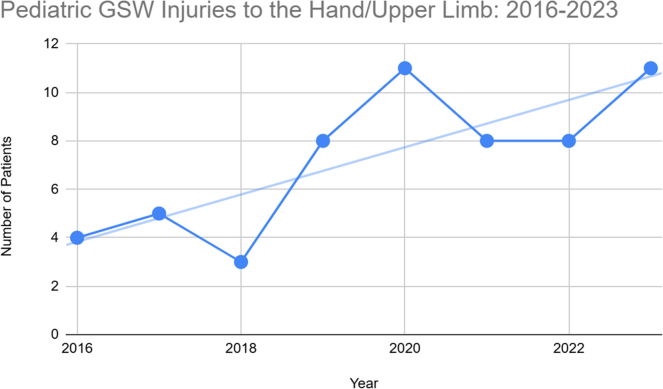
Yearly GSW injuries to the UE presenting to our level-1 trauma center from 2016 to 2023.

Pediatric GSW presentations that received specialized orthopedic evaluation (*n* = 139) increased by 129%, from 21 in 2016 to 48 in 2023 (*τ* = 0.388, *P* = .005). Hand and UL injuries rose by 175%, from 4 in 2016 to 11 in 2023 (*τ* = 0.390, *P* = .224) (Fig. 2).

Most patients were men (79.3%, *n* = 46) with a mean age of 15.2 years (range 6–17 years). Hispanic ethnicity was predominant (65.5%, *n* = 38), significantly higher than the state’s 47.7%. Hispanic population (*P* < .001). African American patients were also overrepresented (6.9% vs 1.8% statewide, *P* < .001), whereas non-Hispanic white patients were underrepresented (17.2% vs 36.5% statewide, *P* < .001). Native American and Asian patients were proportionate to state population levels.

Accidental discharge was the leading cause of injury (41.4%, *n* = 24), followed by assault (37.9%, *n* = 22). Most patients presented to the emergency room (86.2%, *n* = 50), and the most common injury location was the home (26.1%, *n* = 12) (Table 1).

**Table 1:** Demographic Characteristics of Patients With Confirmed Upper-Limb Injuries (*n* = 58)

Category	Subcategory	Number (%)
**Sex**		
	Male	46 (79.3%)
	Female	12 (20.7%)
**Age**	Mean (y)	15.2 (2.61)
**Mechanism of injury**		
	Accidental discharge	24 (41.4%)
	Assault	22 (37.9%)
	Self-inflicted	0 (0%)
	Unknown	12 (20.7%)
**Setting of injury**		
	Home	12 (20.7%)
	Street	10 (17.2%)
	Car/drive-by	9 (15.5%)
	Park	7 (12.1%)
	Party	3 (5.2%)
	Work	1 (1.7%)
	Unknown	16 (27.6%)
**Ethnicity**		
	Hispanic/Latino	38 (65.5%)
	White	10 (17.2%)
	African American	4 (6.9%)
	Native American	2 (3.5%)
	Asian	0 (0%)
	Unknown	4 (6.9%)

**Figure 3:**
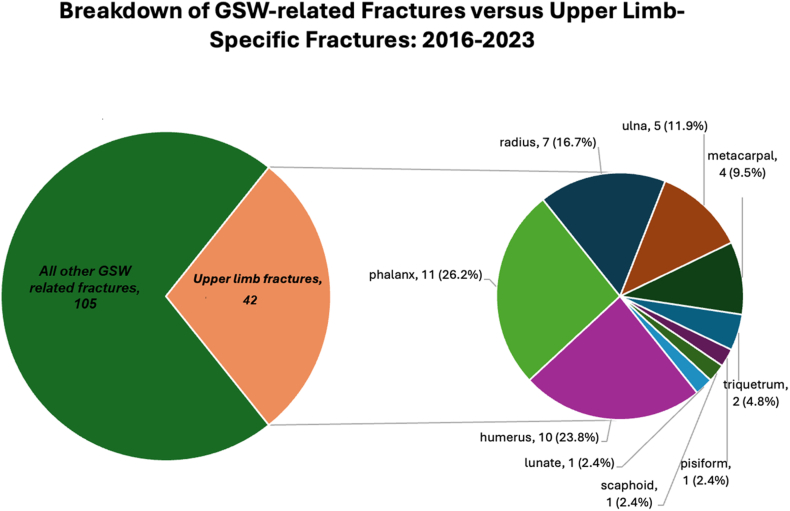
Distribution of fracture patterns of 139 orthopedic consults, with emphasis on upper-extremity fractures (*n* = 42) at our state’s only level-1 trauma center from 2016 to 2023.

Seventy-seven total sequelae were observed among the UL cohort (Table 2). Forty of 58 total patients sustained upper-limb fractures, with 42 total fractures across the phalanges (*n* = 11), humerus (*n* = 10), and radius (*n* = 7) (Fig. 3). Notably, 15 patients experienced peripheral nerve injury, and only 1 patient sustained permanent digit loss as a result of their ballistic injuries (Table 2).

**Table 2:** List of Total Primary and Secondary Sequelae Resulting From GSWs for Patients With Upper-Limb Injuries

Sequelae	Number of cases (% of total sequelae)
Primary sequelae (*n* = 57)	
Fracture (see Fig. 3)	42 (54.5%)
Peripheral nerve injury	15 (19.5%)
Radial	3
Ulnar	4
Median	1
Medial antebrachial cutaneous	1
Anterior interosseus	2
Digital nerves	4
Secondary sequelae (*n* = 20)	
Symptomatic retained foreign body	7 (9.1%)
Symptomatic hardware	3 (3.9%)
Stiffness	4 (5.2%)
Infection	3 (3.9%)
Traumatic amputation	1 (1.3%)
Delayed union/nonunion	1 (1.3%)
Posttraumatic arthritis	1 (1.3%)

There were 57 primary sequelae (fractures or peripheral nerve injuries), and there were 20 secondary sequelae (symptomatic retained foreign body or hardware, stiffness, infection, traumatic amputation, delayed nonunion, posttraumatic arthritis).

Surgical intervention was required in 36 patients (62.1% of UL injuries). Operative management was categorized into early (eg, wound irrigation and debridement, fracture fixation) and late (eg, nerve repair, soft tissue reconstruction) procedures (Table 3). Of the 42 fractures, 22 were treated surgically, with either open reduction internal fixation or intramedullary nailing; therefore, 20 fractures were treated nonoperatively through conservative measures. Twelve patients (33.3%) had other concomitant injuries requiring multidisciplinary surgical specialty care in addition to hand and upper-extremity surgery, notably general surgery (27.8%), otolaryngology (13.9%), and vascular surgery (11.1%). Three infections were reported (9.4%), none involving osteomyelitis. No cases of heterotopic ossification or other osseous complications were noted (Table 2).

**Table 3:** Breakdown of Surgical Management for 36 Total Patients With Upper-Limb Injuries

Surgical Details	Number of Cases
Early/acute intervention (*n* = 59)	
Acute operative irrigation and debridement	22
Open reduction internal fixation	19
Foreign body removal	13
Intramedullary nailing	3
Tendon repair/transfer	2
Late/chronic intervention (*n* = 14)	
Digital/Upper-extremity nerve procedures	11
Neurolysis (5)	
Nerve decompression (3)	
Nerve transfer (1)	
Nerve allograft reconstruction (1)	
Guyon canal release (1)	
Symptomatic hardware removal	2
Revision amputation	1

Eleven of the 15 patients with peripheral nerve injuries underwent delayed nerve procedures for persistent symptoms and functional deficits following their initial injury. Injuries involved a range of peripheral nerves, most commonly the radial and ulnar nerves. Postoperative follow-up demonstrated variable recovery. At the last documented follow-up, 8 of the 11 patients had near full sensory and motor recovery, whereas 3 of the 11 patients required extension of their hand therapy.

Regarding socioeconomic status, 37.9% (*n* = 22) of participants lived in ZIP codes with a median household income below $40,000, which is higher than the proportions observed in the general population of the city (28.5%) and the state (30.1%), in which this study took place. Despite this, only 1 patient in the cohort was uninsured (1.7%).

## Discussion

The high incidence of GSW-related injuries among the pediatric population is a consistent concern both regionally and nationally.^3^^,^^12^ Our data reflect these trends, with 412 total pediatric cases of firearm-related injury presenting during the study period. Nearly 15% of these patients specifically sustained ballistic injuries to the hand and/or UL, illustrating the burden fellowship-trained upper-extremity surgeons carry in managing this patient population. Our study contributes valuable statistics on UE and hand injuries among the pediatric population and adds essential insights from the southwestern US to the national picture concerning pediatric GSWs.

### Upper-limb injuries and demographics

Reported ballistic fracture statistics of the UL in pediatric patients are variable in the literature. Loder and Luster^2^ reported a 48.4% upper-extremity ballistic fracture rate in a 27-year national study, with the fingers being the most commonly affected. Naranje et al^13^ identified a 19% fracture rate among children and adolescents presenting with GSWs at 2 level-1 trauma centers over 9 years, with the humerus being the most frequently involved. Among orthopedic consults, our study found 42 upper-limb fractures, with the majority occurring in the phalanges (26.2%) and humerus (23.8%).

The hand surgery service bears a considerable clinical burden in managing musculoskeletal injuries resulting from firearms at our level-1 trauma institution, as they were consulted for 47% of all patients with ballistic injuries during the 8-year study period. For hand and UE injuries in particular, the hand surgery service, consisting of both plastics and orthopedics-trained hand/UE surgeons, is responsible for all upper-limb trauma and manages the orthopedic burden of the hand and UE subcohort. This rate is higher than in other reports. Lieu et al^14^ found that only 14% of pediatric GSW patients required orthopedic consultation, but emphasized the importance of treating these patients at trauma centers with specialized care. Our findings highlight the substantial burden placed on the hand surgery service in managing these injuries and this service’s critical role in the comprehensive care of pediatric GSW patients. Given the prevalence of UE gunshot-related injuries reported among our cohort, it is ideal that these injuries are managed by a surgeon with specialty training in hand/upper extremity for achieving optimal patient outcomes.

The demographics of pediatric patients sustaining GSWs to the hand and upper extremities in our study were predominantly men (79%), with an average age of 15 years (range 6–17 years). Hispanic and African American patients were overrepresented compared with the state’s overall population demographics (48% and 2%, respectively). Hispanic patients accounted for 66% of cases, whereas African American patients comprised 7%. These findings are consistent with other studies reporting demographic trends in pediatric GSWs. Woodruff et al^10^ observed that 76.7% of pediatric GSW cases involved males, with 68.3% of patients being African American in a Florida-based study. Numerous studies also underscore the overrepresentation of African Americans and males in firearm violence.^2^^,^^14^, ^15^, ^16^, ^17^, ^18^ In our study, ballistic injury rates among the pediatric African American population remain noteworthy (*P* = .001).

### Mechanisms and settings of injury

Our data revealed a wide distribution of injury mechanisms, with accidental discharge (41%) and assault (38%) being the most common causes. This contrasts with findings from Perkins et al,^9^ who reported a different trend from a level-II pediatric trauma center in North Carolina, with 32% of injuries because of accidental discharge and 60% resulting from assault. Interestingly, the setting of injury was unknown in nearly one-quarter (23.9%) of our cases, highlighting potential challenges in obtaining accurate information. Patients may be hesitant to disclose the true nature of their injury, particularly in the presence of law enforcement or because of fear of legal consequences, leading to underreporting or inconsistent narratives.

The location and setting of injury are critical factors to consider when developing preventative strategies for pediatric patients. In our cohort, nearly a quarter of GSW-related injuries occurred in the home (21%), which mirrors findings from Woodruff et al,^10^ in which 28% of pediatric injuries also occurred in the home. This underscores the importance of targeted interventions aimed at improving safety within domestic environments, particularly for at-risk populations. Understanding the context of these injuries is essential for guiding prevention efforts and policy development to reduce the incidence of life-changing injuries among children and adolescents.

Lastly, nearly 40% of patients in our cohort resided in lower socioeconomic ZIP codes, concentrated within inner-city areas with a median household income of $40,000 or less.^19^, ^20^, ^21^ This is notably higher than the surrounding city (29%) and state (30%) averages, emphasizing the disproportionate burden of firearm-related injuries on economically disadvantaged communities. This further underscores the need for targeted public health reforms in these communities, including advocacy efforts, gun safety education in schools, and increased awareness of the impact of firearm violence on children.

### Limitations and future directions

Limitations of our study include the generalizability of the data collected in the southwest to other regions of the country. Although our findings highlight trends in this region, other areas may experience different patterns of firearm-related injuries in terms of severity, prevalence, and demographics. Additionally, the notable number of patients who did not disclose the setting of their injury suggests a reluctance to share information with health care providers. Retrospective studies are inherently limited by the accuracy of recorded data, as they do not benefit from direct patient evaluation. Future research should aim to expand the understanding of pediatric firearm injuries on a national scale. Improvements in data collection through multicenter and prospective studies would enhance the accuracy of documentation and address the limitations of current research.

Gunshot wounds to the upper extremities among the pediatric population remain a critical concern with extensive implications for medical care and public health. We present data from our single-center, 8-year cohort study (2016–2023) on pediatric (<18 years old) GSW injuries to the UL, which includes 58 patients. These injuries increased consistently throughout the study period, suggesting that the upward trend persists beyond what is reported in the literature; however, our data were not statistically significant. Orthopedic surgeons play a crucial role in addressing this epidemic by enhancing treatment protocols and advocating for effective measures to protect children, a role that is likely to remain critical in the coming years as the issue of gun violence continues to challenge our health care system.

## Conflicts of Interest

No benefits in any form have been received or will be received related directly to this article.
