# Supplementary material for: Firearm-Related Upper-Limb Injuries in Children: An 8-Year Single Institution Analysis
Source: J Hand Surg Glob Online. 2026 Feb 26;8(3):100968. doi: 10.1016/j.jhsg.2026.100968 (PMC12955628; doi:10.1016/j.jhsg.2026.100968)
Supplement: Supplementary Table 1 [file mmc1.docx]

**Table A1:**

| ***ICD-10 Code*** | ***Description*** |
| --- | --- |
| X93 | Assault by handgun discharge |
| X94 | Assault by rifle, shotgun, and larger firearm discharge |
| X95 | Assault by other and unspecified firearm and gun discharge |
| X72 | Intentional self-harm by handgun discharge |
| X73 | Intentional self-harm by rifle, shotgun, and larger firearm discharge |
| X74 | Intentional self-harm by other and unspecified firearm and gun discharge |
| W32 | Accidental handgun discharge and malfunction |
| W33 | Accidental rifle, shotgun, and larger firearm discharge and malfunction |
| W34 | Accidental discharge and malfunction from other and unspecified firearms and guns |
